# Common genetic variation in circadian clock genes are associated with cardiovascular risk factors in an African American and Hispanic/Latino cohort

**DOI:** 10.1016/j.ijcha.2021.100808

**Published:** 2021-06-03

**Authors:** Pablo Salazar, Sreenivas Konda, Arvind Sridhar, Zarema Arbieva, Martha Daviglus, Dawood Darbar, Jalees Rehman

**Affiliations:** a Department of Medicine, Division of Cardiology, University of Illinois at Chicago, Chicago, IL, USA; b Division of Epidemiology and Biostatics, School of Public Health, University of Illinois at Chicago, Chicago, IL, USA; c Genomics Core, Research Resources Center, University of Illinois at Chicago, Chicago, IL, USA; d Institute for Minority Health Research, University of Illinois at Chicago, Chicago, IL, USA; e Department of Pharmacology and Regenerative Medicine, The University of Illinois, College of Medicine, Chicago, IL, USA

**Keywords:** Genetics, Cardiovascular Disease, Underrepresented minorities, Ethnicity, Hypertension, Diabetes, Circadian rhythms

## Abstract

Misalignment of the internal circadian time with external physical time due to environmental factors or due to genetic variantion in circadian clock genes has been associated with increased incidence of cardiovascular risk factors. Common genetic variation in circadian genes in the United States have been identified predominantly in European ancestry individuals. We therefore examined the association between circadian clock single nucleotide polymorphisms (SNPs) in *Clock, Cry1, Cry2, Bmal1 and Per3* genes and cardiovascular risk factors in African Americans and Hispanic/Latinos. We analyzed 17 candidate circadian SNPs in 1,166 subjects who self-identified as African-American or Hispanic/Latino and were enrolled in the UIC Cohort of Patients, Family and Friends. We found significant differences in the minor allele frequencies between African American and Hispanic/Latino subjects. Our analyses also established ethnic-specific SNPs that are associated with cardiovascular risk factors. In Hispanic/Latinos, the rs6850524 in *Clock* was associated with increased risk for hypertension, meanwhile rs12649507, rs4864546, and rs4864548 reduced the risk, also rs8192440 (*Cry1*) reduced the risk for type 2 diabetes. In African Americans, the *Clock* rs1801260 and rs6850524 were negatively associated with the presence of obesity; *Bmal1* rs11022775 reduced the risk for dyslipidemia; and the *Cry2* rs2292912 increased the risk for dyslipidemia and diabetes. Genetic variations in candidate circadian-clock genes are associated with risk factors for cardiovascular disease in African-Americans and Hispanic/Latinos. Our findings may help to improve cardiovascular risk assessment as well as better understand how circadian misalignment impacts cardiovascular risk in diverse populations.

## 1 Introduction

Cardiovascular disease (CVD) is the leading cause of death in the United States, and is characterized by marked disparities in its prevalence and health impact across different racial and ethnic groups [1]. African Americans (AAs) and Hispanic/Latinos (H/Ls) have disproportionately higher prevalence of traditional CV risk factors, they also have worse prognosis when compared to the non-Hispanic white populations [2]. While some of the increased CVD risk and prevalence in ethnic minorities can be attributed to socio-economic disparities, recent studies also point toward common genetic variation as key contributors [3], [4], [5]. However, even though ethnic minorities have a greater burden of CV risk factors, they are underrepresented in genetic association studies examining the association between single nucleotide polymorphisms (SNPs) and CV risk. This may in part contribute to the persistence of CV health disparities [6]. Candidate SNPs for diabetes, obesity, hypertension (HTN), and dyslipidemia in subjects of European and Asian descent vary widely from those associated with CV risk factors in AAs and H/Ls [7], [8], [9], [10]; underscoring the importance of ethnic diversity in establishing genetic associations with CVD and CV risk factors.

The disruption of circadian rhythmicity is increasingly being recognized as a major contributor to development of CV risk factors [11]. The master circadian clock is a complex structure located in the suprachiasmatic nucleus of the hypothalamus and generates a near 24-hour endogenous rhythm via transcriptional-translational feedback loops involving the expression of key circadian clock associated genes such as the Circadian Locomotor Output Cycles Kaput gene (*Clock*), the Brain muscle arnt-like gene (isoforms *Bmal 1 and Bmal 2*), the *Period* gene (isoforms *Per1*, *Per2 and Per3*) and the *Cryptochrome* gene (isoforms *Cry1* and *Cry2*). These feedback loops interactions generate the rhythmic oscillation which drives important metabolic and physiological tissue functions [12]. Experimental models have demonstrated that many key physiological variables are influenced by circadian rhythms [13], [14], and that misalignment between the internal circadian time and the external physical time due to environmental or genetic factors are associated with increased sympathetic tone, obesity, HTN, increased insulin resistance, and endothelial dysfunction [15], [16], [17]. Human studies have also demonstrated a clear relationship between circadian disruption – the misalignment of internal circadian time and the external physical time – and the development of metabolic syndrome, obesity and HTN [18]. This misalignment is commonly due to environmental factors such as shift-work, but can also be a result of genetic variants in circadian clock genes such as *Clock*, *Period*, *Bmal* and *Cry* which can increase CV risk [19], [20], [21]. However, current evidence linking variants in circadian genes with susceptibility to CV risk factors is derived from studies conducted in subjects of European and Asian descent. It remains unclear whether common genetic variation in circadian genes are also associated with CV risk factors in ethnic minority populations such as AAs and H/Ls. Here, we explored the association between candidate SNPs in circadian clock genes with key CV risk factors in a cohort of AAs and H/Ls and identified ethnicity-specific SNPs associated with HTN, obesity and type 2 diabetes (T2D).

## 2 Material and methods

### 2.1 Ethics statement

Written informed consent was obtained from all participants under a protocol approved by the University of Illinois at Chicago (UIC) Institutional Review Board (IRB).

### 2.2 Study population

We used the subset of subjects enrolled in the University of Illinois at Chicago (UIC) Cohort of Patients, Family, and Friends for whom blood samples were available for DNA extraction at the time of the analysis (n = 1,166). All individuals were aged 18 or older, and self-identified as being African-American (AA) or Hispanic/Latino (H/L). Demographic characteristics, medical history, resting blood pressure and blood samples for DNA extraction were collected for these subjects individuals > 18 years old..

### 2.3 Sample collection and processing for assessment of SNPs

Blood samples were drawn into one vial. The buffy coat layer containing the white blood cells for DNA extraction was collected from the serum. DNA was extracted using a commercially available kit (Qiagen Puregene, Valencia, CA) and samples were stored in a −80 °C freezer until genotyping. Targeted genotyping was performed using the iPLEX Gold chemistry and MassARRAY System, which includes RS1000 Nanodispenser and MALDI-TOF mass spectrometry analyzer (Agena Bioscience, San Diego, CA). Multiplexes of genotyping assays interrogating each individual SNP of interest were designed using online Assay Design Suite 2.0 (Agena) and manufactured at IDT (Integrated DNA Technologies, Coralville, IA). Each of the assays included gene specific PCR amplification primers and extension primer designed to anneal next to a SNP of interest and to facilitate a single base extension reaction.

DNA samples were processed according to the iPLEX Gold genotyping protocol. Briefly, 2 μl of each DNA sample at concentration ranging from 2 ng/μl to 200 ng/μl was used to amplify the desired region of interest by PCR using gene-specific primers. Next, extension primer annealing reaction was followed by a single base extension to identify the locus-specific allele(s). When primer is extended, dependent upon the template sequence, resulting fragments reflect allele-specific differences in mass. This mass difference allows the data analysis software to differentiate between existing SNP alleles. Purified extension products were deposited to SpectroCHIP arrays using RS1000 Nanodispenser. Spectral analysis was performed using MALDI-TOF mass spectrometer and MassARRAY Analyzer software. Generated spectra were manually reviewed for quality and used for cluster analyses, genotype calling and report generation using the TyperAnalyzer software.

### 2.4 SNP genotyping

We selected a total of 17 polymorphisms for 5 circadian clock associated genes that had been previously reported to be associated with cardiovascular risk factors in populations of European and Asian descent (Supplementary Table 1). Design of the multiplex assays was carried out using the Agena Bioscience Assay Design Suite v2.0. Genotype calls were made by TyperAnalyzer software using the Autoclustering algorithm, with the following parameters, recommended by Agena Bioscience: Specificity 0.9, Magnitude Cutoff 5, Low Mass Sensitivity 0.99, Hetero Sensitivity 0.95, High Mass Sensitivity 0.99, Angle Height, Magnitude SNR, Low Mass Static Limit 15, Hetero Static Limit 15, High Mass Static Limit 15. Genotype calls were manually curated to exclude outliers and final genotyping reports were generated. Thereby, association analysis was performed in the candidate SNPs.

### 2.5 Statistical methods

Descriptive statistics are presented as mean ± standard deviation (SD) for continuous variables and counts (proportions) for categorical variables. We assessed differences between controls and cases using a chi-square test for independence or a 2-sample *t*-test for baseline-demographics variables. Fisher’s exact test was conducted in place of Chi-square test when at least one of the expected sample counts was<5. Genotyping data, assay statistics, and quality control (QC) parameters for the selected samples were derived from peak area data. All quality matrices including statistics of assay and sample performance across the entire dataset were used to exclude poor performing samples and assays from downstream analyses. Individual genotype outputs in a tabular format were combined into a large matrix format for subsequent genetic analysis using PLINK and Golden Helix genome data analyses. Using logistic regression for a binary outcome and multiple regression for a quantitative outcome in PLINK association analysis was performed in both unadjusted and adjusted for age and sex assuming an additive genetic model for all SNPs that passed QC. Multiple comparisons were accounted by using the Bonferroni correction to determine the appropriate significant threshold (0.05/17 = 0.0029).

## 3 Results

### 3.1 Study sample characteristics

The clinical characteristics of the study subjects are shown in Table 1. We included a total of 1,166 individuals, (52% female, 48% male). A total of 601 individuals self-identified themselves as AAs and 565 (48%) as H/Ls with mean age of 52 ± 10.5 and 45 ± 13.8 years respectively. Most of CV risk factors such as T2D (P = 0.27), obesity (P = 0.33), and dyslipidemia (P = 0.46) were evenly distributed, but HTN was significantly more prevalent in AAs (P = 0.001). In the H/Ls group, these results are in line with previous reports, but the prevalence of HTN (57% vs 44%) and dyslipidemia (57% vs 33%) was higher in AAs than previously reported [22].

**Table 1:** The clinical and Demographics Characteristics of the study cohort by race and ethnicity.

Characteristics	African Americans (N = 601)	Hispanic/ Latinos (N = 565)	P-value	Difference in means or proportions (95% CI)	Odds Ratio (95% CI)
**Demographics**					
Age, mean (SD)	52.12 (10.56)	45.15 (13.87)	0.0001	6.97 (5.55–8.39)	–
Female, n (%)	314 (52.25)	338 (59.82)	0.0092	0.08 (0.02–0.13)	0.7348 (0.58–0.93)
**Comorbidities, n (%)**					
Hypertension	340 (57.43)	151 (27.96)	0.0001	0.29 (0.24–0.35)	3.48 (2.71–4.46)
Dyslipidemia	340 (57.43)	187 (35.55)	0.4637	−0.02 (-0.08–0.04)	0.91 (0.71–1.16)
Type 2 Diabetes	125 (21.01)	91 (18.40)	0.2712	0.03 (-0.03–0.09)	1.18 (0.88–1.58)
Obesity	301 (50.08)	267 (47.26)	0.3345	0.03 (-0.03–0.09)	1.12 (0.89–1.41)
Heart Failure	9 (1.69)	35 (5.88)	0.0003	−0.04 (-0.06–0.02)	3.63 (1.73–7.6)
Atrial Fibrillation	30 (5.13)	5 (0.94)	0.0001	0.05 (0.03–0.07)	5.69 (2.19–14.79)
Stroke	11 (2.04)	41 (6.91)	0.0001	−0.05 (-0.03–0.07)	3.57 (1.82–7.02)
PAD*	35 (6.03)	21 (3.91)	0.1041	0.02 (-0.01–0.05)	1.58 (0.91–2.75)
COPD†	17 (3.22)	45 (7.64)	0.0013	0.04 (0.02–0.07)	2.49 (1.41–4.40)
Myocardial infarction	37 (6.22)	23 (4.31)	0.1838	0.02 (-0.01–0.05)	1.47 (0.86–2.51)
BMI, kg/m2‡	31.36 (18.19)	31.06 (7.0)	0.4851	0.31 (0.56–1.89)	–
SBP, mmHg§	124.0 (19.61)	116.6 (17.19)	0.0001	7.45 (5.32–9.58)	–
DBP, mmHg| |	77.19 (12.45)	70.54 (11.15)	0.0001	6.65 (5.29–8.00	–
MAP, mmHg#	92.79 (13.94)	85.88 (12.27)	0.0001	6.91 (5.40–8.42)	–
Cholesterol, mg/dl	177.1 (38.28)	183.0 (38.93)	0.0084	−5.98 (-1.54–10.42)	–

*Peripheral Artery Disease, †Chronic Obstructive Pulmonary Disease, ‡Body Mass Index, §Systolic Blood Pressure, | | Diastolic Blood Pressure, #Mean Arterial Pressure.

### 3.2 Circadian clock SNPs and allele frequencies in ethnic minorities

Two *Per3* SNPS (rs150812083 and rs139315125) and one *Cry1* SNP (rs184039278) were also excluded from bivariate and multivariate statistical analyses because the minor allele could not be identified (Table 2). The remaining 14 circadian clock SNPs exhibited significant differences in the minor allele frequencies (MAFs) across both racial and ethnic groups (Table 3). In the H/L group, the *Clock* gene showed the highest observed frequencies in the rs4864546 SNP (48%) followed by the rs4864548 (46%), and rs12649507 (43%). In the AA group, the highest MAF was found in the *Cry2* rs2292912 (76%) and in the *Clock* rs6850524 (63%). When comparing our findings with the previously reported MAFs primarily based on individuals of European or Asian descent, we found that only rs1801260 (p = 0.0807), rs10002541(p = 0.9364), rs4580704 (p = 0.9915), and rs228697 (p = 0.6917) SNPs in H/L have the same MAF (Supplementary Table 2) as in the previously published literature [23]. Each of the MAF for the 14 SNPs in AA subjects diverged markedly with P-value much lower than 0.05 from those reported on individuals of European or Asian descent [23].

**Table 2:** Basic characteristics for genotyped candidate circadian clock single nucleotide polymorphisms (SNPs).

Gene	SNP	Chromosome	Allele (Major/Minor)	HWE‡ Hispanic/Latinos p-value	HWE African Americans p-value	Literature Data §	
						Allele	MAF
***Clock***	rs1801260	4:55435202	A/G	0.112	1	G	0.2296
	rs4580704	4:55460540	C/G	0.342	0.366	G	0.2776
	rs12649507	4:55514317	G/A	0.193	0.04121	A	0.3574
	rs4864546	4:55537960	G/A	0.7268	0.07825	A	0.4259
	rs4864548	4:55547636	G/A	0.1989	5.75E-09	A	0.3768
	rs10002541	4:55528844	T/C	0.3965	0.2063	C	0.2776
	rs6850524	4:55515830	C/G	0.4625	0.7871	C	0.4453
***Per3***	rs10462020	1:7820623	T/G	1	1	G	0.1206
	rs10462021	1:7837073	A/G	1	1	G	0.1210
	rs150812083 †	1:7809893	C/-	NA	NA	G	0.0034
	rs139315125 †	1:7809900	A/-	NA	NA	G	0.0034
	rs228697	1:7827519	C/G	0.02463	1	G	0.0603
***Cry1***	rs8192440	12:107001328	G/A	0.3686	0.1283	A	0.2103
	rs184039278 †	12:106992962	T/-	NA	NA	G	0.001
***Cry 2***	rs11605924	11:45851540	A/C	0.01014	0.8228	C	0.3259
	rs2292912	11:45856137	C/G	0.01796	0.82	G	0.4996
***Bmal 1***	rs11022775	11:13352217	C/T	0.02272	0.2377	T	0.1873

† no minor allele detected, ‡ Hardy Weinberg Equilibrium, § 1000genome project database. We analyzed 17 polymorphisms in 5 candidate circadian genes. The majority of the studied polymorphisms were *Clock* gene polymorphisms, which had been previously associated with cardiovascular or metabolic diseases in European and Asian populations. Among the 17 polymorphisms, the minor allele was not identified for two polymorphisms in the *Per 3* gene (rs150812083, rs139315125) and one polymorphism in the *Cry 1* gene (rs184039278) in our study population which is why these polymorphisms were subsequently excluded from further analysis. Both groups showed different Hardy Weinberg Equilibrium distributions (HWE) for four polymorphisms (rs228697, rs11605924, rs2292912, rs11022775) in the Hispano/Latino group and two (rs12649507, rs4864548) in the African American group did not satisfy the HWE criteria and they were also subsequently excluded from further analysis.

**Table 3:** Minor allele frequencies (MAFs) for candidate single nucleotide polymorphisms (SNPs) in AAs and H/Ls.

Gene	SNP	H/L Minor allele	Hispanic/Latinos MAF	African Americans MAF	Difference in MAF	P-value
***Clock***	rs1801260	G	0.2002	0.1867	0.0135	<0.0001
	rs10002541	C	0.2761	0.2358	0.0403	<0.0001
	rs4864546	A	0.4829	0.3445	0.1384	<0.0001
	rs12649507	A	0.4351	0.1862	0.2489	<0.0001
	rs4580704	G	0.2778	0.2394	0.0384	<0.0001
	rs6850524	C	0.3799	0.6357	−0.2558	<0.0001
	rs4864548	A	0.4611	0.2138	0.2473	<0.0001
***Per3***	rs10462020	G	0.1768	0.08446	0.09234	<0.0001
	rs10462021	G	0.1772	0.08361	0.09359	<0.0001
	rs228697	G	0.05645	0.01689	0.03956	<0.0001
***Cry1***	rs8192440	A	0.1703	0.1622	0.0081	<0.0001
***Cry2***	rs11605924	C	0.4505	0.103	0.3475	<0.0001
	rs2292912	C	0.4075	0.7632	−0.3557	<0.0001
***Bmal1***	rs11022775	T	0.1418	0.2983	−0.1565	<0.0001

When a SNP satisfies the Hardy Weinberg Equilibrium (HWE) criteria, it is not evolving and average allele frequencies will stay the same across generations which is why SNPs satisfying HWE criteria as best suited for studying association with disease in a given population. The (HWE) was reached for the majority of the circadian clock SNPs (Table 2). Out of 14, four SNPs (*Per3* rs228697, *Cry 2* rs11605924, *Cry 2* rs2292912, *Bmal 1* rs11022775) in the H/Ls group and two *Clock* SNPs (rs12649507 and rs4864548) in the AAs group did not satisfy the HWE criteria and they were therefore excluded from bivariate and multivariate statistical analyses.

### 3.3 Circadian clock SNPs and CV risk factors in ethnic minorities

In AAs 12 SNPs in 5 different circadian clock genes conformed to HWE, and we examined their association with four CV risk factors using a multivariate logistic regression analysis**.** We found that in AAs subjects, *Clock* SNP rs1801260 (OR 0.69, 95% confidence interval [CI], 0.51–0.95; P = 0.024) and rs6850524 (OR 0.74, 95% CI, 0.58–0.97; P = 0.026) were negatively associated with the presence of obesity. The *Bmal1* SNP rs11022775 (OR 0.70, 95% CI 0.52–0.94; P = 0.016) was negatively associated with dyslipidemia. Finally, the *Cry2* SNP rs2292912 was a significant risk factor for the presence of dyslipidemia (OR 1.33, 95% CI 1.01–1.78; P = 0.049) and T2D (OR 1.48, 95% CI 1.08–2.05; P = 0.015). However, none of the results were found to be significant at Bonferroni corrected alpha value. We did not find any association between CV risk factors with the *Clock* SNPs rs4580704, rs4864546, rs1000254, the *Cry2* SNP rs11605924 or the *Per3* and *Cry1* SNPs (Table 4).

**Table 4:** Circadian single nucleotide polymorphism (SNPs) and cardiovascular risk factors in African Americans.

Gene	SNP	Hypertension		Obesity		Dyslipidemia		Type 2 Diabetes	
		OR (95% CI)	P-value	OR (95% CI)	P-value	OR (95% CI)	P-value	OR (95% CI)	P-value
***Clock***	rs1801260	0.8229	0.2243	0.6965	0.02406	0.7357	0.07354	NA	NA
	rs4580704	1.075	0.6158	1.199	0.2011	1.18	0.2522	NA	NA
	rs4864546	1.206	0.1466	0.97	0.8098	0.9915	0.9481	1.279	0.09609
	rs10002541	1.069	0.6479	1.169	0.2765	1.187	0.2364	1.1	0.527
	rs6850524	1.08	0.5595	0.7484	0.02575	0.9747	0.8475	0.8413	0.2624
***Per3***	rs10462020	1.095	0.6883	0.8827	0.5736	0.7929	0.3299	NA	NA
	rs10462021	1.077	0.7446	0.8962	0.6222	0.8081	0.3715	NA	NA
	rs228697	1.82	0.2238	1.04	0.9349	2.127	0.105	NA	NA
***Cry1***	rs8192440	1.17	0.3786	0.8014	0.2047	0.7084	0.06335	NA	NA
***Cry2***	rs11605924	1.267	0.2656	1.45	0.07457	1.42	0.08673	NA	NA
	rs2292912	1.178	0.2725	1.263	0.1077	1.335	0.04985	1.489	0.01474
***Bmal1***	rs11022775	0.9872	0.9273	1.209	0.1721	0.6972	0.01631	0.7471	0.09042

NA, not available.

We performed a multivariate analysis to establish any potential associations between circadian clock gene SNPs and CV risk factors in the H/Ls, for which a total of 7 SNPs in 3 different genes were in HWE (Table 5). The *Clock* rs12649507 SNP (OR 0.78, 95% CI 0.61–0.98; P = 0.0403) and (OR 0.64, 95% CI 0.47–0.86; P = 0.0032) was negatively associated with obesity and HTN respectively; the *Clock* rs4864546 (OR 0.65, 95% CI 0.49–0.89; P = 0.0069) and *Clock* rs4864548 SNPs (OR 0.66, 95% CI 0.49–0.89; P = 0.0057) were negatively associated with HTN. The *Clock* rs6850524 SNP was associated with HTN prevalence (OR 1.35, 95% CI 1.01–1.82; P = 0.04) as well as lower prevalence of dyslipidemia (OR 0.63, 95% CI 0.48–0.85; P = 0.0019). The *Cry1* rs81922440 SNP (OR 0.54, 95% CI 0.34–0.89; P = 0.02) was negatively associated with T2D. After adjusting for multiple testing using the Bonferroni correction, many of these SNPs still remained significant. The *Clock* rs10002541 and the *Per3* s10462021 SNPs were not associated with CV risk factors in H/Ls (Table 5).

**Table 5:** Circadian single nucleotide polymorphism (SNPs) and cardiovascular risk factors in Hispanic/Latinos.

Gene	SNP	Hypertension		Obesity		Dyslipidemia		Diabetes	
		OR (95% CI)	P-value	OR (95% CI)	P-value	OR (95% CI)	P-value	OR (95% CI)	P-value
***Clock***	rs12649507	0.6368	0.003196	0.779	0.04047	1.18	0.21	0.8359	0.29
	rs4864546	0.6596	0.006925	0.819	0.1106	1.19	0.20	0.8846	0.47
	rs4864548	0.6625	0.005695	0.8276	0.1161	1.14	0.3231	0.8482	0.32
	rs10002541	1.364	0.06235	1.103	0.4807	0.74	0.0576	NA	NA
	rs6850524	1.356	0.04412	1.252	0.07752	0.63	0.002	0.9038	0.56
***Per3***	rs10462021	1.024	0.9025	1.216	0.2237	1.28	0.16	NA	NA
***Cry1***	rs8192440	0.9953	0.9804	0.8194	0.213	0.86	0.40	0.5415	0.0168

Considering the identification of *Clock* SNPs with significant positive and negative associations with HTN in H/Ls subjects, we next performed a multivariate analysis between candidate circadian *Clock* polymorphisms and the mean arterial pressure (MAP) in order to estimate the magnitude of impact on BP. Our quantitative trait analysis showed that the risk alleles of *Clock* rs4864546, rs12649507, and rs4864548 SNPs were each associated with a decrease of MAP by ~ 2 mmHg (P < 0.01), whereas the presence of the rs6850524 risk allele (P < 0.01) was associated with an average increase of 2 mmHg in the MAP (Table 6).

**Table 6:** Circadian single nucleotide polymorphism (SNPs) and mean Arterial pressure in Hispanic/Latinos.

Gene	SNP	Allele	N	Regression Coefficient	Standard Error	95% Confidence Interval	T statistic	P value
***Clock***	rs4864546	A	526	−1.957	0.7044	(-3.338,-0.5768)	−2.78	0.0056
	rs12649507	A	547	−2.548	0.6814	(-3.883, −1.212)	−3.74	0.0002
	rs6850524	C	533	2.115	0.718	(0.7073, 3.522)	2.94	0.0034
	rs4864548	A	552	−2.214	0.6767	(-3.54, −0.8877)	−3.27	0.001

## 4 Discussion

In this study we investigated the association between common genetic variation in candidate circadian clock genes and CV risk factors in subjects of African and Hispanic descent. Our finding that distinct circadian clock SNPs are associated with CV risk factors in individuals with varying ethnic or racial background highlights the importance of ensuring the diversity of study population when assessing how circadian misalignment contributes to CV risk.

The MAFs differed between AA and H/Ls groups, thus indicating that genetic propensities for chronobiological patterns and circadian clock associated CV risk factors differ across racial and ethnicities. In H/Ls, the *Clock* rs4864546, rs4864548, and rs12649507, and the *Cry2* rs11605924, and rs2292912 SNPs were the most common. The MAFs of the circadian clock SNPs in H/Ls were similar to those reported in European and Asian populations [23]. In the AAs the most common SNPs in circadian clock genes were in *Cry2* (rs2292912) and *Clock* (rs6850524), with consistently higher MAFs when compared to that reported in European and Asian populations [23], which have been associated with susceptibility to abdominal obesity and T2D [24], [27]. The less frequent alleles were *Per3* rs228697 and rs10462021, and showed slightly lower values when compared with other racial or ethnic populations [26], [28] (Table 2).

Hypertension remains one of the most important risk factors for CVD therefore early diagnosis and appropriate treatment is crucial to improve CV outcomes [29]. In our study we found that several circadian SNPs which had been previously linked with metabolic diseases in subjects with European and Asian ancestry, demonstrated a significant association with HTN in H/Ls. In this group, carrying the *Clock* SNP rs6850524 increased the risk for HTN by 1.35-fold. Interestingly, this *Clock* SNP also reduced the prevalence of dyslipidemia in H/Ls subjects. This dual association of selectively increasing the risk for one disease while reducing it for another disease has been previously reported for this *Clock* SNP, as it has been linked in humans to obesity in Chinese populations and reduced prevalence of nonalcoholic fatty liver disease in European ancestry population [24], [30], but it has not been associated with HTN or dyslipidemia and is has also not been previously associated with such dual effects within a single population.

We also found that the *Clock* SNPs rs12649507, rs4864546, and 4,864,548 (all upstream transcript variants) were negatively associated with the presence of CV risk factors in H/Ls. The *Clock* rs4864546 and *Clock* rs4864548 SNPs have been associated with obesity and T2D in Asian populations [24], [31] but the association with HTN is new. Nevertheless, this possible protective effect of selected *Clock* SNPs in preventing HTN is compatible with findings described in animal models, in which *Clock* knockout mice consistently exhibit a lower BP and hypotension phenotypes when compared to wild type (WT) mice [32]. One prior study, conducted in subjects of European descent demonstrated a positive relationship between the *Clock* SNP rs1801260 and HTN [33], but this SNP was not associated with HTN in either H/Ls or AA subjects in our study. The exact mechanism by which *Clock* variants impact BP regulation remains unclear, but animal models have suggested that they can modulate renal vasoconstrictors, heart rate variability, sodium excretion and endothelial function [15], [16], [32], [34], [35]. Understanding the interaction between the circadian system and BP regulation is not only of interest in terms of assessing risk for HTN but circadian clock-oriented therapies can lead to significant clinical benefits. For example, advanced chronic kidney disease patients experience a disruption of the physiological BP and suffer from marked nocturnal HTN, which can be controlled by the use of nocturnal hemodialysis as well as nocturnal doses of anti-hypertensives which lead to significantly improved outcomes [36], [37], [38].

We also studied the association between *Clock* variants and obesity and found that the *Clock* SNP rs12649507 (upstream transcript variant) in H/Ls and the *Clock* SNPs rs1801260 (downstream transcript variant) and rs6850524 (upstream transcript variant) in AAs, were all associated with a reduced prevalence of obesity. The *Clock* gene and its relationship with obesity has been extensively studied in animal models and in humans. *Clock* mutant mice demonstrate disruption in metabolic rates and feeding rhythms causing hyperphagia and obesity when compared to WT mice [39]. In human studies with subjects of Asian ancestry, the *Clock* rs6850524 has been associated with a reduced prevalence of obesity [24], consistent with what we found in AAs. However, this same variant is associated with a 1.26-fold increased prevalence of being overweight in a Latin American population that was self-reported as having European ancestry [25]. The *Clock* rs1801260 was also associated with reduced susceptibility to obesity in a European ancestry population, consistent with our findings [40]. The rs12649507 variant has not previously been associated with obesity, but a prior report conducted in subjects of European descent showed that this variant is associated with a favorable dietary profile characterized by an increase in polyunsaturated fatty acids intake, but the underlying causes and its potential impact on obesity remain unclear [41].

Approximately 19% of AAs and 12% of H/Ls over the age of 20 years have been diagnosed with T2D, with a 77% and 66% higher risk respectively when compared with non-Hispanic white Americans [42]. In our study, we identified 2 SNPs associated with T2D. In the H/Ls group, the *Cry1* rs8192440 (coding sequence variant) reduced susceptibility to T2D, whereas in a Northern Sweden population this SNP was associated with seasonal-dependent fasting glucose values, with lower values during light-seasons and higher values during dark-seasons [43]. On the other hand, we found that AAs carrying the *Cry2* SNP rs2292912 was associated with 1.48-fold increased susceptibility to T2D, and this finding is consistent with prior reports in subjects of Asian descent [27]. The *Clock* rs6850524 (upstream transcript variant) in H/Ls and the *Bmal1* rs11022775 (upstream transcript variant) in AAs were both associated with reduced susceptibility to dyslipidemia, whereas the *Cry2* SNP rs2292912 was associated with a significant 1.33-fold increased risk for dyslipidemia in AAs, but there is no prior report of Cry2 SNP association with dyslipidemia.

Many of the SNPs we studied demonstrated a different genotype-phenotype association when compared to prior reports of subjects with predominantly European and Asian ancestry. These results support the notion that genetic variants associated with diseases identified in one racial or ethnic group cannot be necessarily extrapolated to other races and ethnicities.

Our findings should be interpreted in the context of several considerations. First, the designation of race or ethnicity relied on self-report and our cohort likely represents a genetically diverse and heterogeneous group in a large US metropolitan city. Future studies could employ a genetic ancestry analysis to further define the association between circadian clock gene variations and genetic ancestry of individuals. We cannot exclude the possibility that discrepancies between self-identification and genetic ancestry could confound some of the findings. However, our findings could still be very relevant for clinical applications in similar large US metropolitan patient populations who predominantly self-report their race or ethnicity. Second, we correlated circadian clock gene SNPs with susceptibility to CV risk factors but we did not directly investigate the circadian rhythm or sleep patterns in our cohort nor did we study the mechanisms by which these variants impact HTN, obesity, T2D and dyslipidemia. Future studies with extensive chronobiological characterization using actigraphy and sleep monitoring as well as mechanistic and physiological studies examining the distinct molecular functions of these genetic variants may provide important insights into the underlying mechanisms by which circadian gene SNPs modulate CV risk in ethnic minorities. Third, the majority of the candidate SNPs did not survive Bonferroni correction (except for those in H/Ls). However, the Bonferroni correction is considered overly conservative for datasets with low levels of linkage disequilibrium between SNPs.

To date, around 90% of the genetic data extracted from genome wide association studies (GWAS) is derived from populations with a European ancestry, and efforts to expand the ethnic diversity in genetic association studies has resulted in greater inclusion of Asians but subjects of African and Hispanic ancestry continue to be under-represented [44]. In light of the emerging recognition that the circadian clock is not only a critical modulator of metabolic and CV physiology and common genetic variants are strongly correlated with CV risk factors in subjects of European and Asian ancestry, we determined the relationship of candidate circadian clock SNPs in AAs and H/Ls. Our study provides novel insights into the relationship between circadian clock gene variants and CV risk factor traits in ethnic minorities. Our findings emphasize the importance of studying circadian rhythms and circadian genetics in under-represented minority populations. In order to define the specific contributions of circadian clock gene SNPs in increasing the risk of hypertension or other cardiovascular risk factors, it is necessary to perform GWAS studies in populations with ethnic and racial diversity.

## Declaration of Competing Interest

The authors declare that they have no known competing financial interests or personal relationships that could have appeared to influence the work reported in this paper.

## References

[1] Graham G. (2015). Disparities in Cardiovascular Disease Risk in the United States. Curr Cardiol Rev..

[2] Carnethon MR, Pu J, Howard G, Albert MA, Anderson CAM, Bertoni AG, et al. Cardiovascular Health in African Americans: A Scientific Statement From the American Heart Association. Vol. 136, Circulation. 2017. 393–423 p. 10.1161/CIR.0000000000000534 29061565

[3] Cambien F., Tiret L. (2007). Genetics of cardiovascular diseases: From single mutations to the whole genome. Circulation.

[4] Arnett D.K., Baird A.E., Barkley R.A., Basson C.T., Boerwinkle E., Ganesh S.K. (2007). Relevance of genetics and genomics for prevention and treatment of cardiovascular disease: A scientific statement from the American Heart Association Council on Epidemiology and Prevention, the Stroke Council, and the Functional Genomics and Translational. Circulation.

[5] Kathiresan S., Srivastava D. (2012). Genetics of human cardiovascular disease. Cell.

[6] Mensah G.A., Jaquish C., Srinivas P., Papanicolaou G.J., Wei G.S., Redmond N. (2019). Emerging Concepts in Precision Medicine and Cardiovascular Diseases in Racial and Ethnic Minority Populations. Circ. Res..

[7] Williams Amy A.L., Jacobs Suzanne S.B.R., Moreno-Macías H., Huerta-Chagoya A., Churchhouse C., Márquez-Luna C. (2014). Sequence variants in SLC16A11 are a common risk factor for type 2 diabetes in Mexico. Nature.

[8] Franceschini N., Carty C.L., Lu Y., Tao R., Sung Y.J., Manichaikul A. (2016). Variant discovery and fine mapping of genetic loci associated with blood pressure traits in Hispanics and African Americans. PLoS ONE.

[9] Beecham A.H., Wang L., Vasudeva N., Liu Z., Dong C., Goldschmidt-Clermont P.J. (2016). Utility of blood pressure genetic risk score in admixed Hispanic samples. J. Hum. Hypertens..

[10] Deo R.C., Reich D., Tandon A., Akylbekova E., Patterson N., Waliszewska A. (2009). Genetic differences between the determinants of lipid profile phenotypes in African and European Americans: The Jackson Heart Study. PLoS Genet..

[11] Takeda N., Maemura K. (2011). Circadian clock and cardiovascular disease. J. Cardiol..

[12] Jagannath A., Taylor L., Wakaf Z., Vasudevan S.R., Foster R.G. (2017). The genetics of circadian rhythms, sleep and health. Hum. Mol. Genet..

[13] Albrecht U. (2012). Timing to Perfection: The Biology of Central and Peripheral Circadian Clocks. Neuron.

[14] Takeda N., Maemura K. (2015). The role of clock genes and circadian rhythm in the development of cardiovascular diseases. Cell. Mol. Life Sci..

[15] Viswambharan H., Carvas J.M., Antic V., Marecic A., Jud C., Zaugg C.E. (2007). Mutation of the circadian clock gene Per2 alters vascular endothelial function. Circulation.

[16] Curtis A.M., Cheng Y., Kapoor S., Reilly D., Price T.S., FitzGerald G.A. (2007). Circadian variation of blood pressure and the vascular response to asynchronous stress. Proc Natl Acad Sci U S A..

[17] Bray M.S., Shaw C.A., Moore M.W.S., Garcia R.A.P., Zanquetta M.M., Durgan D.J. (2008). Disruption of the circadian clock within the cardiomyocyte influences myocardial contractile function, metabolism, and gene expression. Am J Physiol - Hear Circ Physiol..

[18] Morris C.J., Yang J.N., Scheer F.A.J.L. (2012). The impact of the circadian timing system on cardiovascular and metabolic function. Prog. Brain Res..

[19] Saxena R., Voight B.F., Lyssenko V., Burtt N.P., de Bakker P.I.W., Chen H. (2007 Jun 1). Genome-Wide Association Analysis Identifies Loci for Type 2 Diabetes and Triglyceride Levels. Science.

[20] Scott E.M., Carter A.M., Grant P.J. (2008). Association between polymorphisms in the Clock gene, obesity and the metabolic syndrome in man. Int J Obes..

[21] Zeggini E., Weedon M.N., Lindgren C.M., Timothy M., Elliott K.S., Lango H. (2007). Multiple type 2 diabetes susceptibility genes following genome- wide association scan in UK samples. Science.

[22] Mozaffarian D, Benjamin EJ, Go AS, Arnett DK, Blaha MJ, Cushman M, et al. Heart Disease and Stroke Statistics—2016 Update. Vol. 133, Circulation. 2016. 38–360 p. 10.1161/CIR.0000000000000350 26673558

[23] Auton A., Abecasis G.R., Altshuler D.M., Durbin R.M., Bentley D.R., Chakravarti A. (2015). A global reference for human genetic variation. Nature.

[24] Ye D., Cai S., Jiang X., Ding Y., Chen K., Fan C. (2016). Associations of polymorphisms in circadian genes with abdominal obesity in Chinese adult population. Obes Res Clin Pract..

[25] Sookoian S., Gemma C., Gianotti T.F., Burgueño A., Castaño G., Pirola C.J. (2008). Genetic variants of Clock transcription factor are associated with individual susceptibility to obesity. Am. J. Clin. Nutr..

[26] Hida A., Kitamura S., Katayose Y., Kato M., Ono H., Kadotani H. (2014). Screening of clock gene polymorphisms demonstrates association of a PER3 polymorphism with morningness-eveningness preference and circadian rhythm sleep disorder. Sci. Rep..

[27] Kelly M.A., Rees S.D., Hydrie M.Z.I., Shera A.S., Bellary S., Hare P.O. (2012). Circadian Gene Variants and Susceptibility to Type 2 Diabetes : A Pilot Study. PLoS ONE.

[28] Ebisawa T., Uchiyama M., Kajimura N., Mishima K., Kamei Y., Katoh M. (2001). Association of structural polymorphisms in the human period3 gene with delayed sleep phase syndrome. EMBO Rep..

[29] Lawes CMM, Hoorn S Vander, Rodgers A, Society I. Global burden of blood-pressure-related disease, 2001. Lancet 2008 May 3;371(9623):1513-8. 10.1016/S0140-6736(08)60655-8 18456100

[30] Sookoian S., Castaño G., Gemma C., Gianotti T.F., Pirola C.J., Sookoian S. (2007). Common genetic variations in CLOCK transcription factor are associated with nonalcoholic fatty liver disease. World J. Gastroenterol..

[31] Uemura H., Katsuura-Kamano S., Yamaguchi M., Arisawa K., Hamajima N., Hishida A. (2016). Variant of the clock circadian regulator (CLOCK) gene and related haplotypes are associated with the prevalence of type 2 diabetes in the Japanese population. J. Diabetes.

[32] Zubera A.M., Centenoa G., Pradervandb S., Nikolaevaa S., Maquelina L., Cardinauxa L. (2009). Molecular clock is involved in predictive circadian adjustment of renal function. Proc Natl Acad Sci U S A..

[33] Kolomeichuk S.N., Makeeva I.V., Topchieva L.V., Korneva V.A., Nemova N.N. (2011). Association of T3111C Polymorphism in 3 ’ Untranslated Region of the Clock Gene with the Risk of Essential Arterial Hypertension and Coronary Artery Disease in the Russian Population (Residents of Karelia). Gentika..

[34] Anea C.B., Zhang M., Stepp D.W., Simkins G.B., Reed G., Fulton D.J. (2009). Vascular disease in mice with a dysfunctional circadian clock. Circulation.

[35] Nikolaeva S., Pradervand S., Centeno G., Zavadova V., Tokonami N., Maillard M. (2012). The circadian clock modulates renal sodium handling. J. Am. Soc. Nephrol..

[36] Culleton B, Walsh M, Quinn R, Donnelly S, Friedrich M, Kumar a. Effect of frequent nocturnal hemodialysis vs conventional hemodialysis. J Am Med Assoc. 2007;298(11):1291–9. 10.1001/jama.298.11.1291 17878421

[37] Boggia J., Li Y., Thijs L., Hansen T.W., Kikuya M., Björklund-Bodegård K. (2007). Prognostic accuracy of day versus night ambulatory blood pressure: a cohort study. Lancet.

[38] Hermida R.C., Ayala D.E., Mojón A., Fernández J.R. (2011). Decreasing sleep-time blood pressure determined by ambulatory monitoring reduces cardiovascular risk. J. Am. Coll. Cardiol..

[39] Turek F.W., Joshu C., Kohsaka A., Lin E., Ivanova G., McDearmon E. (2005). Obesity and metabolic syndrome in circadian Clock mutant nice. Science.

[40] Scott E.M., Carter A.M., Grant P.J. (2008). Association between polymorphisms in the Clock gene, obesity and the metabolic syndrome in man. Int J Obes (Lond)..

[41] Dashti H.S., Follis J.L., Smith C.E., Tanaka T., Cade B.E., Gottlieb D.J. (2015). Habitual sleep duration is associated with BMI and macronutrient intake and may be modified by CLOCK genetic variants. Am. J. Clin. Nutr..

[42] Chow E.A., Foster H., Gonzalez V., McIver L.S. (2012). The disparate impact of diabetes on racial/ethnic minority populations. Clin Diabetes..

[43] Renström F., Koivula R.W., Varga T.V., Hallmans G., Mulder H., Florez J.C. (2015). Season-dependent associations of circadian rhythm-regulating loci (CRY1, CRY2 and MTNR1B) and glucose homeostasis: the GLACIER Study. Diabetologia.

[44] Popejoy A.B., Fullerton S.M. (2016). Genomics is failing on diversity. Nature.

