# Supplementary material for: Common genetic variation in circadian clock genes are associated with cardiovascular risk factors in an African American and Hispanic/Latino cohort
Source: Int J Cardiol Heart Vasc. 2021 Jun 3;34:100808. doi: 10.1016/j.ijcha.2021.100808 (PMC8188044; doi:10.1016/j.ijcha.2021.100808)
Supplement: Supplementary data 1 [file mmc1.docx]

**SUPPLEMENTAL MATERIAL**

**Supplementary Table 1. Basic characteristics for genotyped candidate circadian clock single nucleotide polymorphisms (SNPs)**

| **Gene** | **SNP** | **Chromosome** | **Allele**  **(Major/Minor)** | **HWE**‡  **Hispanic/Latinos**  **p-value** | **HWE**  **African Americans**  **p-value** | **Literature Data** § | |
| --- | --- | --- | --- | --- | --- | --- | --- |
|  |  |  |  |  |  | **Allele** | **MAF** |
| ***Clock*** | rs1801260 | 4:55435202 | A/G | 0.112 | 1 | G | 0.2296 |
|  | rs4580704 | 4:55460540 | C/G | 0.342 | 0.366 | G | 0.2776 |
|  | rs12649507 | 4:55514317 | G/A | 0.193 | 0.04121 | A | 0.3574 |
|  | rs4864546 | 4:55537960 | G/A | 0.7268 | 0.07825 | A | 0.4259 |
|  | rs4864548 | 4:55547636 | G/A | 0.1989 | 5.75E-09 | A | 0.3768 |
|  | rs10002541 | 4:55528844 | T/C | 0.3965 | 0.2063 | C | 0.2776 |
|  | rs6850524 | 4:55515830 | C/G | 0.4625 | 0.7871 | C | 0.4453 |
|  |  |  |  |  |  |  |  |
| ***Per 3*** | rs10462020 | 1:7820623 | T/G | 1 | 1 | G | 0.1206 |
|  | rs10462021 | 1:7837073 | A/G | 1 | 1 | G | 0.1210 |
|  | rs150812083 † | 1:7809893 | C/- | NA | NA | G | 0.0034 |
|  | rs139315125 † | 1:7809900 | A/- | NA | NA | G | 0.0034 |
|  | rs228697 | 1:7827519 | C/G | 0.02463 | 1 | G | 0.0603 |
|  |  |  |  |  |  |  |  |
| ***Cry 1*** | rs8192440 | 12:107001328 | G/A | 0.3686 | 0.1283 | A | 0.2103 |
|  | rs184039278 † | 12:106992962 | T/- | NA | NA | G | 0.001 |
|  |  |  |  |  |  |  |  |
| ***Cry 2*** | rs11605924 | 11:45851540 | A/C | 0.01014 | 0.8228 | C | 0.3259 |
|  | rs2292912 | 11:45856137 | C/G | 0.01796 | 0.82 | G | 0.4996 |
|  |  |  |  |  |  |  |  |
| ***Bmal 1*** | rs11022775 | 11:13352217 | C/T | 0.02272 | 0.2377 | T | 0.1873 |

† no minor allele detected, ‡ Hardy Weinberg Equilibrium, § using data from the 1000genome project database (<https://www.internationalgenome.org/data/>) as a literature reference

**Supplementary Table 1**

We analyzed 17 polymorphisms in 5 candidate circadian genes, Table 2. The majority of the studied polymorphisms were *Clock* gene polymorphisms, which had been previously associated with cardiovascular or metabolic diseases in European and Asian populations. Among the 17 polymorphisms, the minor allele was not identified for two polymorphisms in the *Per 3* gene (rs150812083, rs139315125) and one polymorphism in the *Cry 1* gene (rs184039278) in our study population which is why these polymorphisms were subsequently excluded from further analysis. Both groups showed different Hardy Weinberg Equilibrium distributions (HWE) for four polymorphisms (rs228697, rs11605924, rs2292912, rs11022775) in the Hispano/Latino group and two (rs12649507, rs4864548) in the African American group did not satisfy the HWE criteria and they were also subsequently excluded from further analysis.

**Supplementary Table 2**. Comparison of MAF in H/L and AA with the MAF reported in the Literature.

| **Gene** | **SNP** | **Minor**  **allele** | **Literature Review MAF** | **Hispanic/Latinos MAF** | **P-**  **value** | **African Americans**  **MAF** | **P-value** |
| --- | --- | --- | --- | --- | --- | --- | --- |
| ***CLOCK*** | rs1801260 | G | 0.2296 | 0.2002 | 0.0807 | 0.1867 | <0.0001 |
|  | rs10002541 | C | 0.2776 | 0.2761 | 0.9364 | 0.2358 | <0.0001 |
|  | rs4864546 | A | 0.4259 | 0.4829 | 0.0067 | 0.3445 | <0.0001 |
|  | rs12649507 | A | 0.3574 | 0.4351 | 0.0002 | 0.1862 | <0.0001 |
|  | rs4580704 | G | 0.2776 | 0.2778 | 0.9915 | 0.2394 | <0.0001 |
|  | rs6850524 | C | 0.4453 | 0.3799 | 0.0014 | 0.6357 | <0.0001 |
|  | rs4864548 | A | 0.3768 | 0.4611 | 0.0001 | 0.2138 | <0.0001 |
| ***PER 3*** |  |  |  |  |  |  |  |
|  | rs10462020 | G | 0.1206 | 0.1768 | 0.0005 | 0.08446 | <0.0001 |
|  | rs10462021 | G | 0.121 | 0.1772 | 0.0005 | 0.08361 | <0.0001 |
|  | rs228697 | G | 0.0603 | 0.05645 | 0.6917 | 0.01689 | 0.0013 |
| ***CRY 1*** |  |  |  |  |  |  |  |
|  | rs8192440 | A | 0.2103 | 0.1703 | 0.0114 | 0.1622 | <0.0001 |
| ***Cry 2*** |  |  |  |  |  |  |  |
|  | rs11605924 | C | 0.3259 | 0.4505 | <0.0001 | 0.103 | <0.0001 |
|  | rs2292912 | C | 0.4996 | 0.4075 | <0.0001 | 0.7632 | <0.0001 |
| ***Bmal 1*** |  |  |  |  |  |  |  |
|  | rs11022775 | T | 0.1873 | 0.1418 | 0.0019 | 0.2983 | <0.0001 |

Note. P-values are generated using one-sample Z test for proportions with the literature review MAF as the Null value.
